# Total knee arthroplasty in patients with Klippel Trenaunay syndrome and knee osteoarthritis: A case report and a literature review

**DOI:** 10.1097/MD.0000000000037000

**Published:** 2024-01-26

**Authors:** Jiaxi Li, Guangshui Lv, Zhiyuan Han, Xin Xin

**Affiliations:** a Shandong Wendeng Orthopedic Hospital, Weihai, China; b Shanghai University of Traditional Chinese Medicine, Shanghai, China.

**Keywords:** arthroplasty, case report, Klippel Trenaunay syndrome, knee osteoarthritis, spinal muscular atrophy, total knee arthroplasty, vascular malformation

## Abstract

**Introduction:**

Klippel Trenaunay syndrome (KTS) is a rare congenital disorder characterized by wine staining, varicose veins, bone hypertrophy, and soft tissue hyperplasia. KTS usually occurs at birth, early infancy or childhood. The rarity of disease makes it difficult to calculate its incidence rate. However, few studies report the incidence rate of 2 to 5 cases per 100 thousand. Furthermore, evidence demonstrates that KTS is more prevalent among males compared to females.

**Case presentation:**

An elderly male aged 67, was admitted to the hospital for chronic pain in his left knee. An outpatient physical examination reveals a significantly thicker left lower limb accompanied by multiple varicose veins. The right lower limb was 2 cm short on the opposite side, and the right foot was stunted with high arch deformity. The entire body was covered in a red grape globus, which faded after pressing. He was diagnosed with KTS. We performed TKA for him after blood coagulation examination. The patient recovered well after the operation. He was followed up for 1 year, The patient is in good condition and satisfied with the operation.

**Conclusion:**

For patients with KTS, total knee arthroplasty is an effective surgical procedure to treat arthritis. However, some risks must be considered, and appropriate surgical preparation must be undertaken.

## 1. Introduction

Klippel Trenaunay syndrome (KTS) is a rare congenital disorder characterized by wine staining, varicose veins, bone hypertrophy, and soft tissue hyperplasia.^[1]^ KTS usually occurs at birth, early infancy or childhood. The rarity of disease makes it difficult to calculate its incidence rate. However, few studies report the incidence rate of 2 to 5 cases per 100 thousand.^[2,3]^ Furthermore, evidence demonstrates that KTS is more prevalent among males compared to females.^[3]^

KTS often occurs at birth and childhood, about 95% of patients with KTS have limb hypertrophy. Bone and soft tissue hypertrophy can cause various symptoms, such as uneven limb length, secondary pelvic tilt, joint contracture, and so forth. Asymmetry in the lower limbs and abnormal bone growth leads to knee osteoarthropathy.

The etiology remaines unclear until recently when evidence has emerged that there was a genetic basis for this sporadic disorder. Genes that encode pathological angiogenic factors and cause vascular dysmorphogenesis explaining the molecular bases of this syndrome have been identified. Several angiogenic genes were identified but 1 gene, the AGGF1 (formerly VG5Q) gene, was seen in mutations involving patients diagnosed with KTS. Furthermore, this syndrome was also noted to have overlapping clinical features linked with the “overgrowth syndromes” in which genetic mutations along somatic lines were identified. These involved the PI3K enzyme which forms part of the phosphoinositide 3-kinase pathway which is encoded by the PIK3CA-gene. This enzyme mediates embryonic cellular growth in-utero and diseases involved in this pathway, are classified as members of the PIK3CA-related overgrowth syndrome.^[4]^

The wine staining are regarded as the most common vascular cutaneous malformation in KTS, seen in 98%. These are abnormal ectatic capillaries in the papillary dermis, with the capillary walls being very thin. Varicose veins occur in 72% of patients with KTS,^[5,6]^ the prominent feature being the persistent (embryonic) lateral vein present in 56% of patients,^[5]^ which can be considered as a pathognomonic feature. There are significantly large valveless truncal veins presenting as enormous varicosities, but many other anomalies may exist, such as compressive fibrous bands, aneurysmal dilatation, duplication, hypoplasia, atresia and aplasia. Due to venous stasis in these large valveless veins, deep vein thrombosis (DVT) and associated pulmonary embolism are well known complications of KTS. Lymphatic malformations, which are more common than expected, occurring in 11% of KTS patients. They consist of dilated vessels filled with clear proteinaceous fluid, but they do not connect to normal lymphatic vessels lying in cutaneous and subcutaneous tissue.^[7]^ The lymphatic system has a very close developmental, structural and functional relationship with the venous system,^[8]^ and plays an important role in the symptomatology and progress of these patients. Extensive venous networks extend to spread across viscera of the pelvis and spinal cord. Visceral vascular malformations can be seen in the liver, bladder, rectum, retroperitoneum and pericardium. Patients can therefore present with a variety of symptoms including internal hemorrhage and rectal bleeding.^[4]^

KTS can not be cured at present, therefore, the aims of treatments on KTS are mainly including the management of pain and the control of exercise volume. There are surgical and nonsurgical interventions to treat symptoms. Nonsurgical options include sclerotherapy, laser therapy, and compression therapy, as well as medical management, which includes anticoagulation, pain medications, and anti-inflammatory drugs.^[9–11]^ Surgical options include, but are not limited to, surgical vein ligation, vein stripping, vein resection, epiphysiodesis, tibial osteotomy, arthroplasty, and debulking, Additionally, affected limbs occasionally are amputated to lower cardiac output and myocardial stress in order to prevent heart failure.^[12,13]^

Knee Osteoarthritis and disparity in leg length are common orthopedic effects of KTS. Limb length discrepancies can be appropriately managed with heel inserts or compensatory shoes to avoid scoliosis as long as discrepancies are <1.5 cm. However, if the discrepancy in limb length is >2 cm, surgical intervention should be considered in the form of osteotomy or epiphysiodesis by the appropriately train edorthopedic surgeon.^[14]^ KTS-associated arthropathy is commonly monoarticular because the disease is usually localized to 1 extremity. Our patient presented with degenerative arthropathy of the knee, which is the most commonly affected joint.^[9]^ Besides, our patient suffered from infantile spinal muscular atrophy (SMA). Patients with SMA are usually accompanied by muscle contracture and limited activity caused by muscle weakness, resulting in increased pain, osteoporosis, and limb fracture, which significantly complicates surgery and prognosis.

One of the rarest forms of severe arthritis involves a unilateral lower extremity vascular malformation combined with the ipsilateral knee joint. In this case, total knee arthroplasty (TKA) is full of risks. Baskerville^[15]^ reported that 16% of KTS patients developed deep venous thromboembolism, and 14% of them developed pulmonary emboli. There is a risk of uncontrolled bleeding in KTS. Therefore, standard practice does not recommend orthopedic surgery.

Based on a literature review, limited arthroplasty cases in KTS are available. So far, 5 cases involving the knee and 4 cases involving the hip have been reported.^[9,16–23]^ In one case, rapid bleeding occurred during the operation, and the operation was forced to stop and ended in failure.^[20]^

## 2. Case report

**Figure 1.**
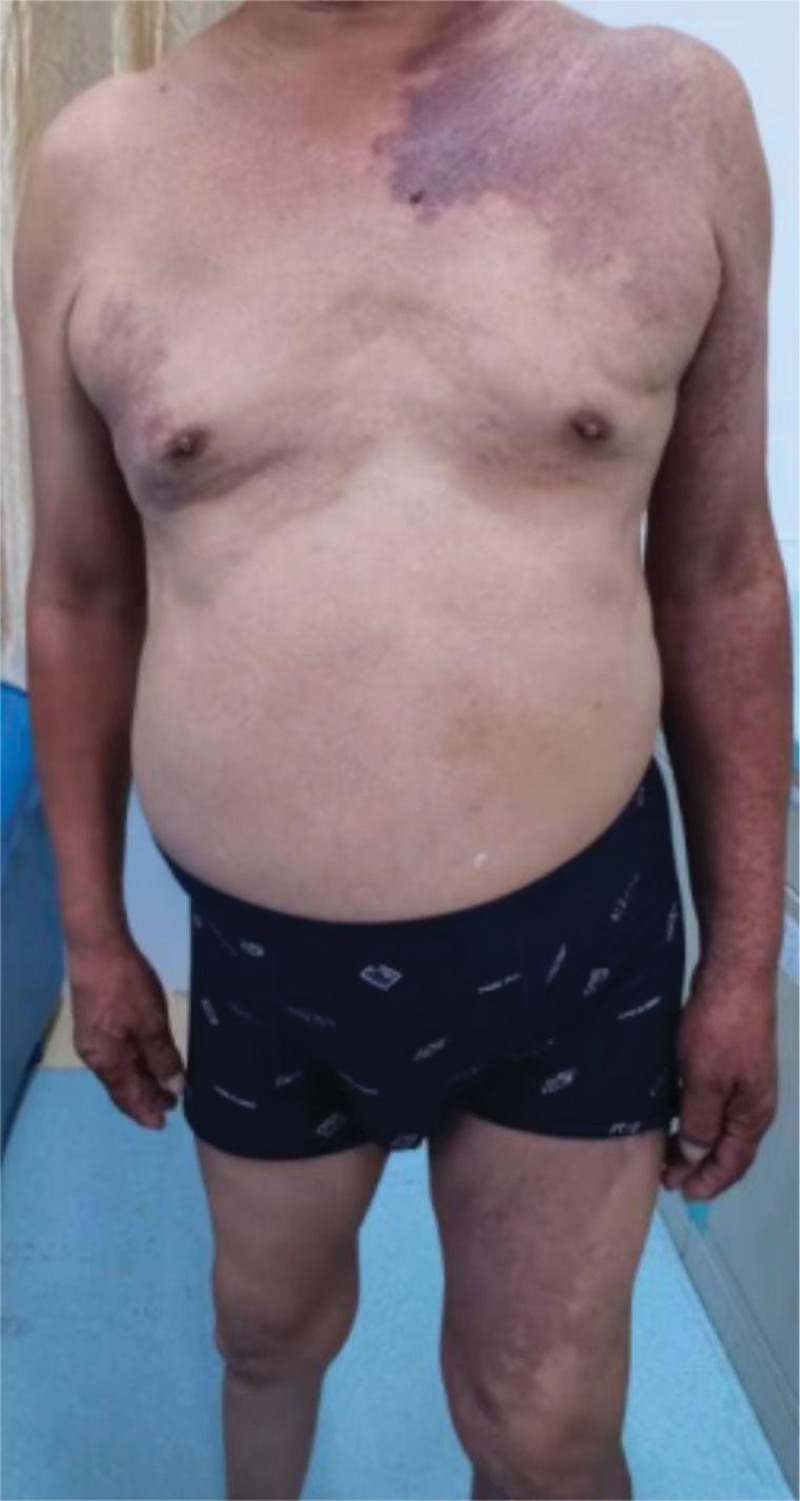
The entire body was covered in a red grape globus.

**Figure 2.**
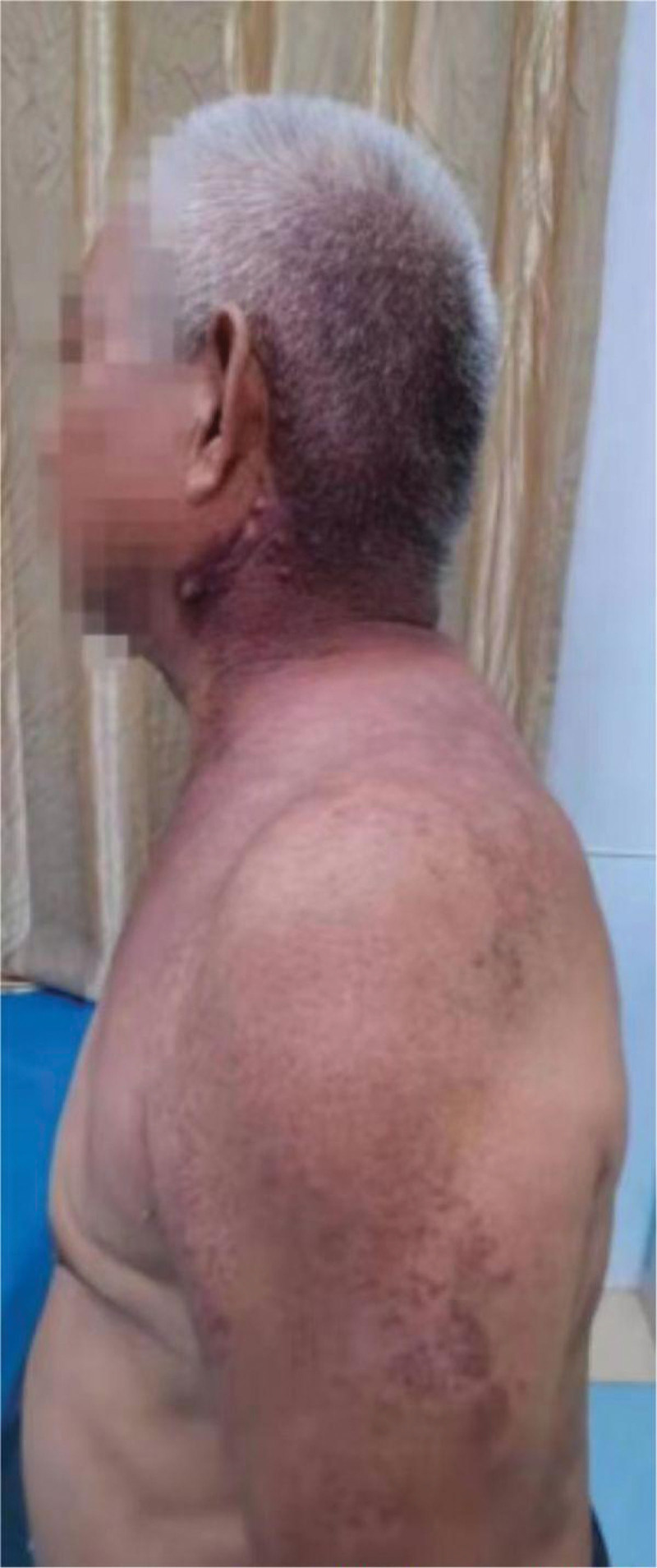
The entire body was covered in a red grape globus.

**Figure 3:**
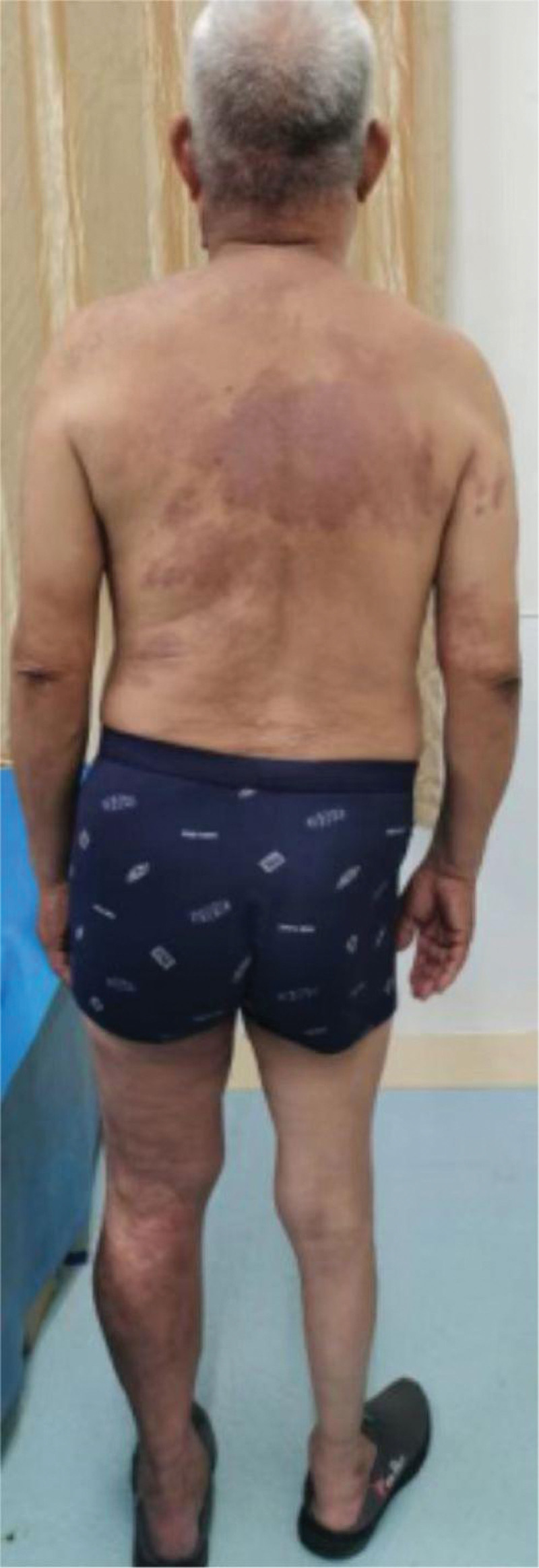
. The entire body was covered in a red grape globus.

An elderly male aged 67 (patient personal privacy information has been removed), was admitted to the hospital for chronic pain in his left knee. An outpatient physical examination reveals a significantly thicker left lower limb accompanied by multiple varicose veins. The right lower limb was 2 cm short on the opposite side, and the right foot was stunted with high arch deformity. The entire body was covered in a red grape globus, which faded after pressing (Figs. 1–3). He was diagnosed with KTS and knee osteoarthritis, and upon inquiry, it was found that he also suffers from SMA, and he does not have a family genetic history of related diseases.

**Figure 4.**
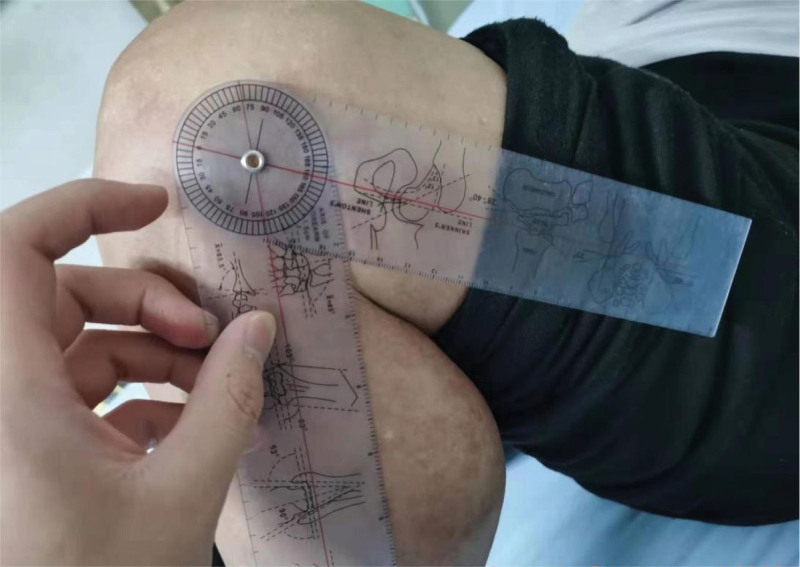
Patient knee joint range of motion.

**Figure 5.**
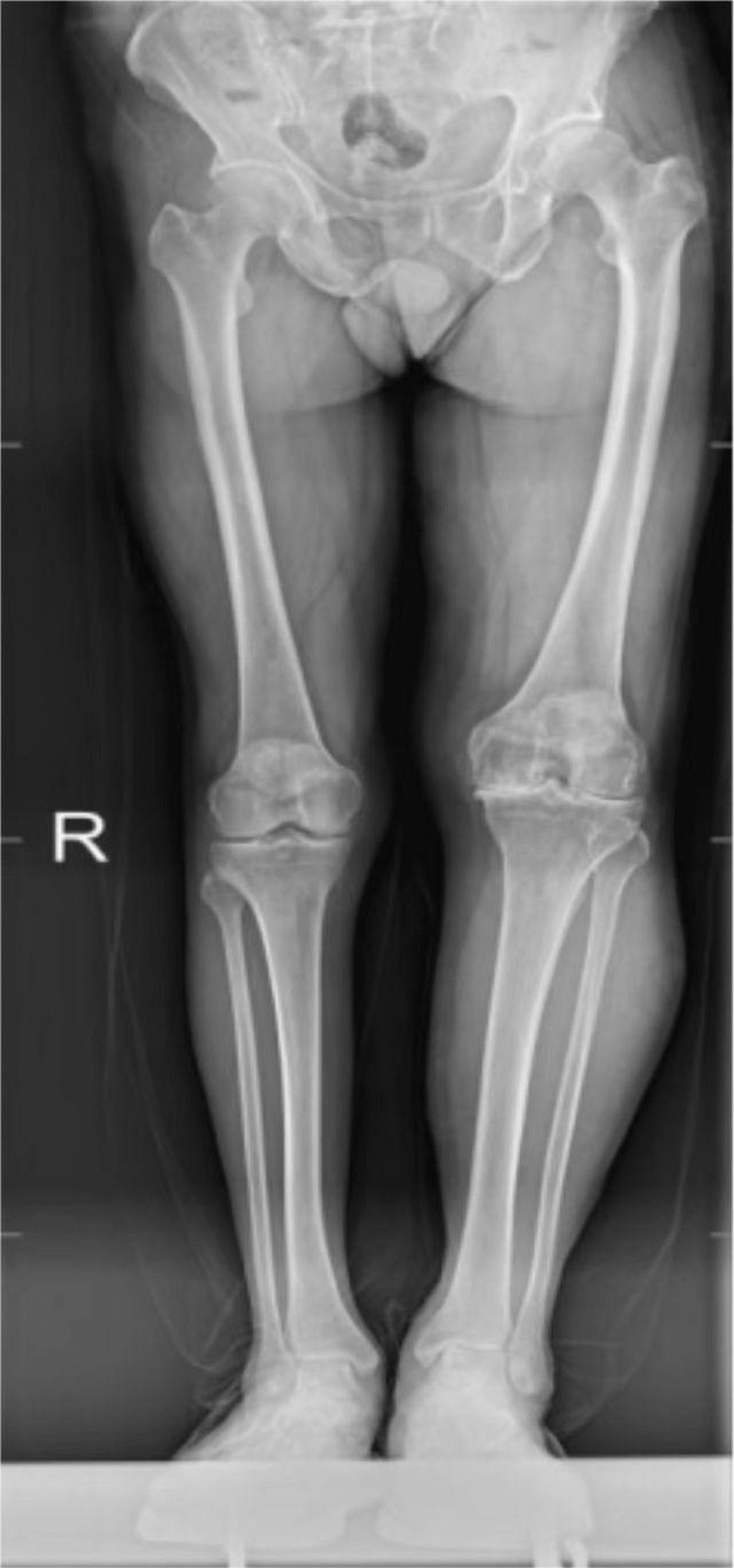
Radiological examination of the left knee showed severe degenerative osteoarthritis, hypertrophic square femoral condyle and subchondral bone sclerosis.

**Figure 6.**
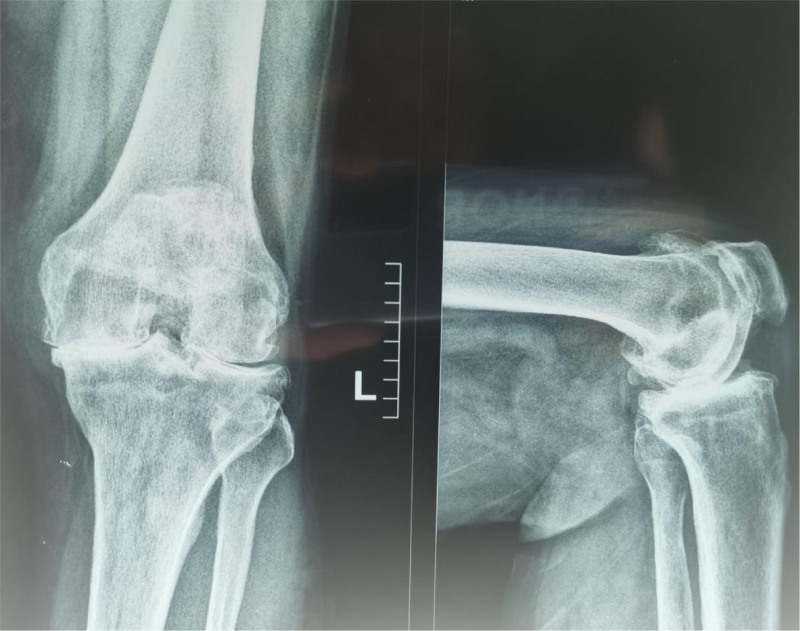
Radiological examination of the left knee showed severe degenerative osteoarthritis, hypertrophic square femoral condyle and subchondral bone sclerosis.

**Figure 7:**
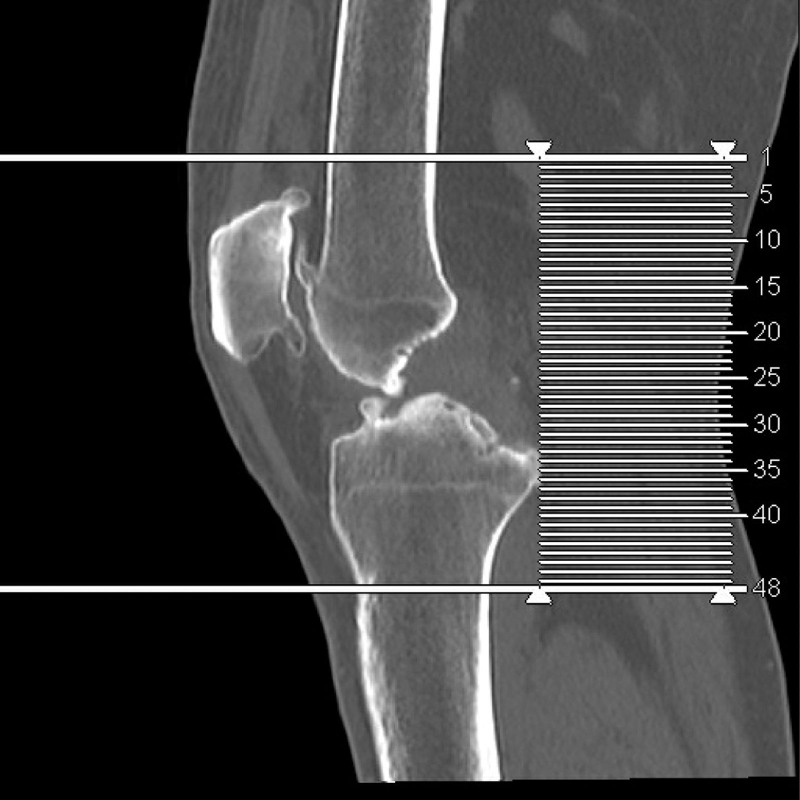
. Radiological examination of the left knee showed severe degenerative osteoarthritis, hypertrophic square femoral condyle and subchondral bone sclerosis.

Further examination revealed that the straightening angle of the left knee was only 20°, the flexion angle could only reach 68° (Fig. 4). The right knee joint could be improved in terms of its functional ability. Extensor dorsalis muscle strength V of the left lower extremity was normal. The extensor dorsalis muscle strength IV of the right lower limb, the pulse of the dorsalis pedis artery of both lower limbs were good, and the ends of both lower limbs could have feeling, blood supply and activity. Radiological examination of the left knee showed severe degenerative osteoarthritis, hypertrophic square femoral condyle and subchondral bone sclerosis (Figs. 5–7).

After many visits and failed non-surgical treatments (including pain relief drugs, Chinese medicine and crutches), we discussed the treatment plan with the patient. The patient wished to undergo a joint replacement, even if he had knew that the risk of total knee replacement failure was high.

A TKA was planned. In addition to medical and cardiac clearance, we obtained a preoperative consultation with the vascular surgery and plastic surgery departments. This workup included a angiogram, which demonstrated 1 area of high-flow arteriovenous malformations. And the preoperative coagulation index were also high: plasma-d dimer: 0.63 µg/mL; plasma fibrinogen determination: 6.68 g/L; ESR: 81 mm/h. Following consideration of the severe bleeding tendency of KTS and the possibility of venous thrombosis, peripheral vascular doctors were consulted, and allogeneic blood 3U was arranged.

The operation was performed under combined spinal anesthesia. A tourniquet was used during the procedure. Several small vascular malformations that were encountered during dissection were cauterized using bipolar radiofrequency and saline solution without complication. The tissues had a hemosiderin-laden appearance, as might be seen in a patient with hemophilia. Considering the bone hypertrophy and bone deformation caused by KTS, we prepared large knee prosthesis and restrictive prosthesis before operation. After osteotomy, we measured the model and found that the fit of the common prosthesis was good. A German link bone cement prosthesis was implanted successively, followed by patella denervation. Two synovial samples were taken intraoperatively for frozen section histology; None of them showed signs of acute inflammation. Permanent pathology specimens were also taken; The bone and synovial tissue removed during the operation were replaced by vascular hyperplasia with lymphoplasmacyte exudates. Pathological examination further revealed a visible vascular mass with iron phagocytosis, which corresponding with the examination results of hemangiomas in KTS.^[21]^

The tourniquet was used for 80 minutes, and intravenous injections of tranexamic acid were administered 30 minutes before the operation, prosthesis implantation, and after suturing for joint cavity perfusion. Next, the drainage tube was placed, and approximately 600 mL of the drain was collected. After 24 hours, the drainage tube was removed.

The patient postoperative course was uncomplicated since there were no apparent signs of anemia and the patient did not require blood transfusions. Moreover, low molecular weight heparin was administered postoperatively to prevent venous thrombosis.

The patient began continuous passive exercise on the other day and started walking using a walker. Because the patient opposite limb had weak SMA muscle strength, the patient right lower limb with swelling and pain was difficult to walk after surgery, and the length of bilateral limbs was different. We prepared compensatory shoes for him, and invited a rehabilitation doctor to consult, and formulated a quadriceps exercise plan for him. In addition, the patient was instructed to use an intermittent air pump device to prevent venous thrombosis and perform ankle pump exercises. Ultrasonography of both lower limbs was performed 5 days after surgery, and no signs and symptoms of DVT were found. On the same day, the ROM was 0° to 90° and his wound healed well.

**Figure 8.**
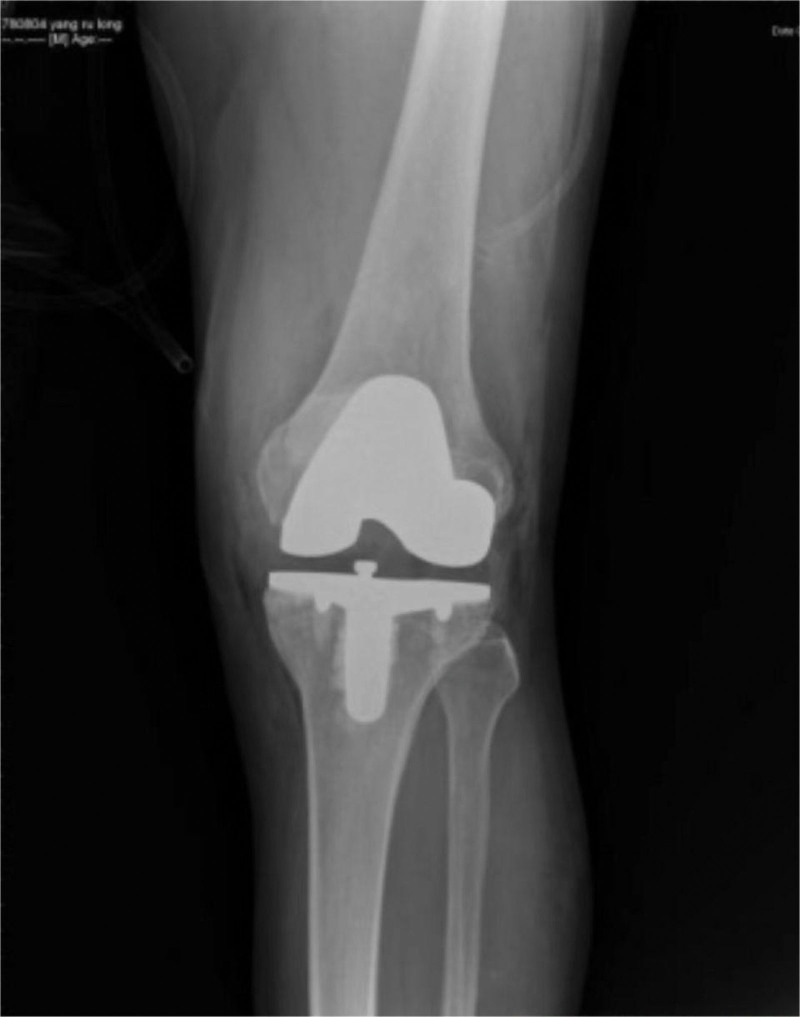
X-ray after 1 yr shows prosthesis are in good condition.

**Figure 9:**
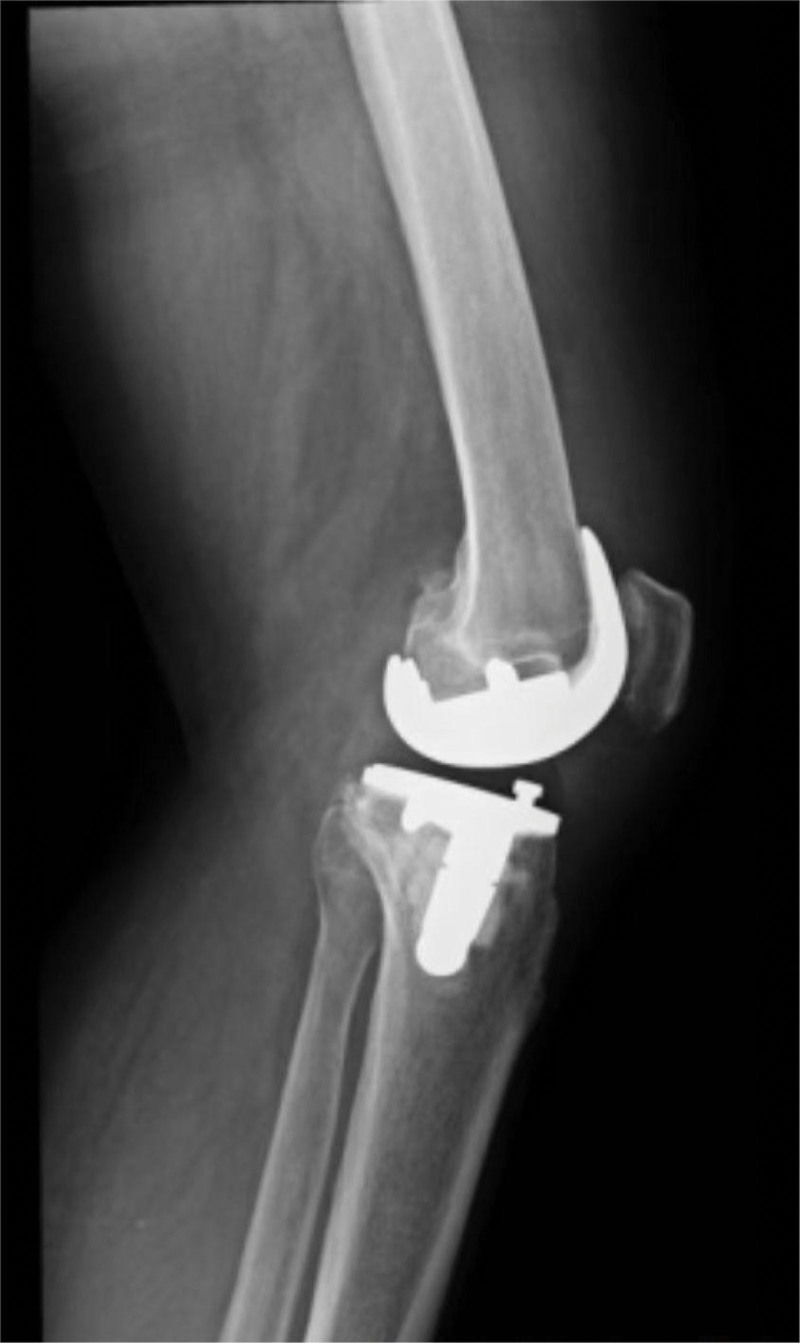
. X-ray after 1 yr shows prosthesis are in good condition.

A year later, the patient reported a significant reduction in knee pain during follow up examination, X-ray showed the prosthesis are in good position (Figs. 8 and 9). In addition, the patient was able to walk normally and had found a job. Furthermore, the patient was able to travel by congested bus without external support. The patient expressed satisfaction with the postoperative outcomes.

## 3. Discussion

Patients with vascular malformation should be managed similarly to those with more common varieties of chronic venous disorders with appropriate consideration of potentially extenuating circumstances such as deep venous agenesis.^[14]^ The latter may make the remaining superficial veins a very important egress for lower extremity outflow. Patients presenting with symptoms of chronic venous disease should be initiated on a regimen of compression and elevation. Compression stockings should extend from above the affected area to the digits and should be fitted to the individual limb. The correct pressure may vary for the patient but typically ranges from 20 to 40 mm Hg. However, compliance to this garment can be difficult in the pediatric population with rapidly growing limbs along with other social factors. Low-dose aspirin should be considered as there is limited benefit to pain and swelling in patients with vascular malformations.^[24]^ If symptoms are refractory to compression alone, intermittent pneumatic compression can be an useful adjunct in lower extremity edema mobilization.^[25]^ In this case, our patient used compression stockings to relieve the pain caused by vascular malformation, but the effect was little. After surgery, we used intermittent air pump device to relieve the pain caused by vascular malformation, and we prescribed aspirin for the patient after discharge to prevent DVT and reduce the pain and swelling caused by vascular malformation.

In patients with KTS, it is difficult to perform a TKA because abnormal bone growth and blood vessels in the affected limb pose a risk of uncontrollable bleeding. However, the only treatment for osteoarthritis caused by KTS is joint replacement.

Compared with the 3 patients who underwent TKA in the literature, our case shows some significant differences and similarities.^[9,17,18]^ The differences between these 3 cases and ours include age and comorbidity. Our patient was significantly older at 67 years of age compared with patients aged 35, 38, and 30 years in those literatures. Our patient had a combined history of SMA, while the other cases did not have any noteworthy comorbidities. The cases were similar with regard to exhausting non-surgical options, using a cemented prosthesis, and limb-length discrepancies that were noted on physical examination.

Patients with KTS have been reported to have a high risk of postoperative complications, wound complications, cardiovascular complications, and infection. In these cases, bleeding and embolism are likely to occur without adequate preoperative preparation. Therefore, a multidisciplinary consultation is necessary prior to an operation to assess its risk. However, we encountered varicose veins and vascular masses during surgery, which were burned to stop bleeding. Although the patient did not receive a postoperative blood transfusion, it is necessary to prepare allogeneic blood before the operation.

Catreetal^[21]^ pointed out that tourniquets were useful to avoid massive bleeding during knee surgery. However, they also encountered varices and hemangiomas during the operation.

## 4. Conclusion

Few studies have been conducted on joint replacement techniques in patients with KTS. Due to abnormal bone hypertrophy and vascular malformation, it is risky to operate on people with KTS. In this case report, we hope to provide some reference to prepare patients with KTS for surgery.

For patients with this vascular malformation undergoing joint replacement, we want to suggest the following:

1. Appropriate vascular research should be carried out prior to surgery to determine the severity of the vascular malformation.
2. If venous embolism is detected before surgery, multidisciplinary consultation is necessary, particularly with vascular surgeons.
3. As patients with KTS have a severe bleeding tendency, a sufficient amount of allogeneic blood must be prepared before the operation to avoid massive bleeding.
4. Tranexamic acid should be administered during the perioperative period to reduce bleeding, and anticoagulant activity should be monitored to prevent deep vein thrombosis.

In addition, special care must be taken to ensure sterility since these patients are more vulnerable to infections. Furthermore, long-term monitoring is necessary because the progression is unpredictable. Apparently, the other side of the patient body had weak muscles. Hence, postoperative rehabilitation exercises are essential for the follow-up to overcome muscle weakness.

## Author contributions

**Formal analysis:** Zhiyuan Han.

**Methodology:** Guanshui Lv, Zhiyuan Han.

**Resources:** Guanshui Lv, Zhiyuan Han.

**Supervision:** Xin Xin.

**Validation:** Xin Xin.

**Writing – original draft:** Jiaxi Li.

**Writing – review & editing:** Jiaxi Li.
